# *Cicer arietinum* extract as antitumor and protective agent against Ehrlich Solid Carcinoma-bearing mice

**DOI:** 10.1186/s12906-025-05061-z

**Published:** 2025-09-02

**Authors:** Amany Ahmed Sayed, Mahmoud Salah Abdullah, Amr Ahmed WalyEldeen, Rasha Mohamed Samir Sayed, Refaat M. Gabre, Sherif Abdelaziz Ibrahim, Hebatallah Hassan

**Affiliations:** 1 Department of Zoology, Faculty of Science, Cairo University, Giza, 12613 Egypt; 2 Department of Biotechnology, Faculty of Science, Cairo University, Giza, 12613 Egypt; 3 Department of Pathology, Faculty of Medicine, Aswan University, Aswan, 81528 Egypt

**Keywords:** Ehrlich solid carcinoma, *Cicer arietinum*, Protective, Anticancer, Antioxidant, Apoptosis, Pyroptosis

## Abstract

**Background:**

Cancer remains a significant global health challenge. Several plant-derived compounds have garnered the attention of cancer research for their anticancer effects. Chickpea (*Cicer arietinum*) is one of the top nutritious legumes with promising chemoprotective effects. We previously demonstrated that *Cicer arietinum* extract (CAE) has antitumor activity in vitro. Therefore, herein, we aimed to extend our findings and investigate CAE’s preventive and therapeutic antitumor effects in vivo using a murine Ehrlich solid carcinoma (ESC) model.

**Methods:**

Thirty-six female mice were divided into six groups: healthy controls, untreated ESC-bearing mice, CAE pre-treated groups (low, moderate, and high doses), and CAE post-treated group administered after tumor establishment. Tumor size, oxidative stress markers, antioxidant enzyme activities, and the expression of apoptotic (*Casp3)-* and pyroptotic *(Gsdmd*)-related genes were assessed. Finally, the immunohistochemical staining for the anti-apoptotic Survivin expression was assessed across different mice groups.

**Results:**

Our data indicate that pre-treatment with moderate and high doses of CAE, as well as post-treatment, significantly inhibited tumor growth by more than 40% relative to untreated ESC-bearing controls. Histopathological analysis showed notable improvements in muscle tissue structure in CAE-treated samples. Mechanistically, CAE exerted its chemopreventive and therapeutic effects *via* the alleviation of oxidative stress by a significant enhancement of the antioxidant enzyme activities and increased *Gpx4* mRNA expression, accompanied by a reduction of MDA and NO levels. Furthermore, CAE attenuated survivin expression, while dramatically boosting the expression of apoptotic *Casp3* and pyroptotic *Gsdmd* markers, particularly in the post-treatment group.

**Conclusion:**

These findings suggest that CAE is a promising anticancer agent with protective effects against ESC.

**Graphical Abstract:**

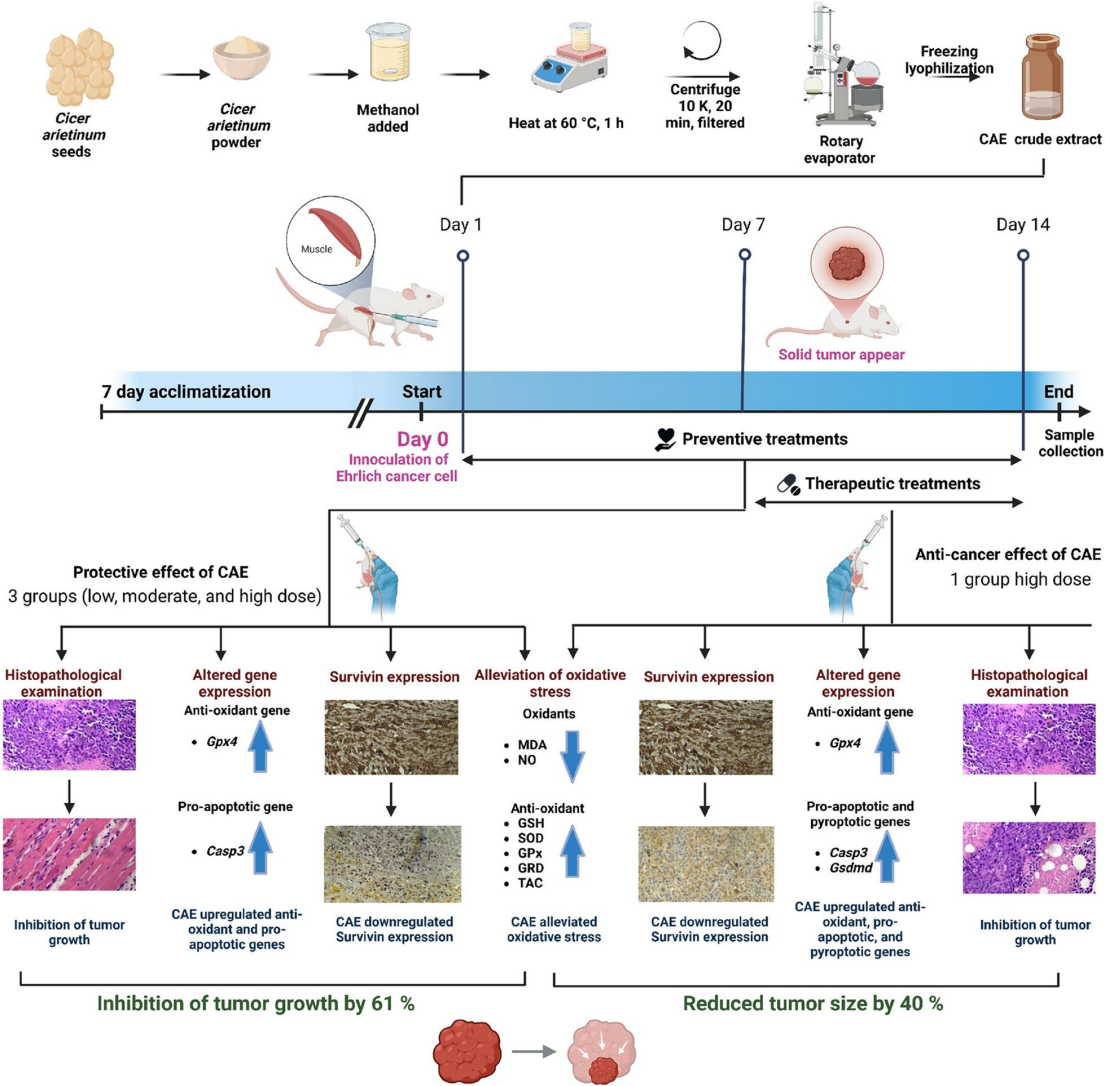

## Introduction

Cancer is considered a significant threat and a potentially life-threatening illness characterized by uncontrolled cell growth. The incidence and mortality rates associated with cancer are on the rise continually [1, 2]. This disease has become a grave concern for scientists worldwide, presenting significant challenges in terms of management [3]. The development of cancerous cells involves multiple stages, namely initiation, promotion, and progression [4]. Factors such as an imbalanced diet, hormonal fluctuations, persistent infections, inflammation, and tobacco use play a crucial role in the initiation of cancer [5]. Despite numerous treatment options available, cancer continues to be the second most prevalent cause of mortality worldwide, inflicting immense devastation [6, 7]. Diverse approaches, including stem cell transplantation, chemotherapy, radiotherapy, immunotherapy, and surgery, have been employed in the effort to combat cancer.

Chemotherapy and radiotherapy continue to be the primary conventional treatments administered for cancer patients. These therapies aim to reduce tumor size and eliminate cancer cells at primary and metastatic locations [8]. The ideal chemotherapeutic agent for treating cancer would effectively target tumor cells while sparing normal cells. However, many traditional anticancer medications lack specificity, leading to unintended toxic effects and patient discomfort [9]. The utilization of naturally derived or synthetically modified dietary phytochemicals for cancer prevention and therapeutic interventions is referred to as complementary and alternative medicine (CAM). Therefore, the urgent need for the development of natural therapeutic options with reduced adverse effects is essential for the prevention and/or suppression of cancer.

Chickpeas are a valuable source of essential macronutrients, including proteins (18.3–25%), complex carbohydrates (54.60–60.40%), and dietary fibers, both soluble (1.23–1.38%) and insoluble (14.1–23.2%). Additionally, they contain lipids (1.12–6.8%) and are abundant in vital micronutrients such as vitamins and minerals (1.94–2.41%). Chickpeas also comprise bioactive compounds, including oligosaccharides like ciceritol and raffinose, phenolic compounds such as formononetin, genistein, and flavonol kaempferol, as well as isoflavones like biochanin A and various phospholipids [10–12].

Numerous pathological factors play an important role in the onset and/or advancement of cancer; among them is the excessive production of reactive oxygen species (ROS), which induces oxidative insult to cells [13]. Numerous phytochemicals, including flavonoids, terpenoids, and steroids, exhibit antitumor potential due to their antioxidant properties that can scavenge free radicals and prevent tumor induction [14]. The methanolic crude extract of chickpea *Cicer arietinum*, the natural agent present in legumes, comprises multiple biochemical constituents. These compounds may exhibit enhanced efficacy in cancer prevention compared to their isolated counterparts, primarily due to their combined additive and synergistic effects, as previously demonstrated [15]. Since *Cicer arietinum* is regarded as a safe extract, as no abnormal clinical symptoms were observed at a dosage of 5000 mg/kg in rat body weight, it consequently exhibits minimal or no adverse effects compared to chemical or radiation-based therapies [16]. Further, *Cicer arietinum* possesses notable antitumor potential, due to its diverse bioactive constituents, as we have previously shown [17]. The extract is rich in isoflavones, including genistein, daidzein, biochanin A, and formononetin, along with a range of phytochemicals such as flavonoids, terpenoids, and steroids [17, 18]. These compounds, working in concert, exert antioxidant activities with free radical scavenging capabilities, which collectively contribute to their potential antitumor efficacy [19–22]. Our research group has previously investigated the anti-tumor effects of *Cicer arietinum* extract *(CAE) in vitro* using the triple-negative breast cancer cell lines MDA-MB-231 and 4T1. We showed that CAE exhibited a significant inhibitory effect on cell viability, colony formation, and cell adhesion. Additionally, CAE attenuated the epidermal growth factor (EGF)-induced p44/42 MAPK signaling pathway, a crucial regulator for cell survival. Notably, treatment with CAE induced morphological transformation in TNBC cells, shifting from a spindle-shaped, fibroblast-like structure to an epithelial-like phenotype. These findings highlight the potential of CAE as a promising anticancer agent against highly aggressive breast cancer subtypes [17].

Additionally, several in vitro studies have highlighted the significant anticancer potential of *Cicer arietinum* through diverse mechanisms, emphasizing its role as a promising natural therapeutic agent. Isolated bioactive compounds of *Cicer arietinum*, such as peptides [23], lectin [24], protease inhibitor [25] and C-25 protein [26] have exhibited notable antiproliferative effects against human cancer cell lines derived from endometrial, breast, prostate, and oral cancers, respectively. Moreover, Chickpea-derived agglutinin [27] and isoflavonoids, including isoflavone [28–30], and coumestrol [28] have been shown to induce apoptosis in various breast cancer cells. In vivo studies further support these findings, demonstrating that dietary inclusion of chickpeas reduces tumor incidence and inflammatory biomarkers in a colon cancer mouse model [31]. Chickpea extracts have also exhibited robust anti-inflammatory effects in a DSS-induced colitis model [23] and immunomodulatory properties in cisplatin-induced immunosuppressed mice [32]. Interestingly, *Cicer arietinum* has also emerged as a promising drug delivery carrier, enhancing anticancer efficacy against A549 lung cancer cells compared to inorganic vanadium [33]. Collectively, *Cicer arietinum* offers a multifaceted approach to cancer treatment by exerting antiproliferative, pro-apoptotic, anti-inflammatory, and immunomodulatory effects across various cancer models. These findings underscore its potential as a natural therapeutic agent for targeted cancer therapy and drug development.

Ehrlich Solid Carcinoma (ESC) is a rapidly growing tumor model commonly used in cancer research. It derives from a spontaneously occurring mammary adenocarcinoma in mice. ESC is a valuable model for investigating the impact of anti-cancer therapies on tumor growth and metastasis, as it mimics numerous clinical, physiological, and biological features of cancer cachexia [34–36]. Thus, in the current study, we explored the effect of the CAE crude extract against mice model inoculated with ESC in vivo using mice model.

## Materials and methods

### Crude *Cicer arietinum* extract (CAE) preparation

This methanolic extract was prepared following the same protocol as described in our previous study [16, 37]. Where HPLC analysis uncovered its major bioactive constituents, including (1) Formononetin, the most abundant, followed by (2) Genistein, (3) Biochanin A, and (4) Daidzein. In brief, 100 g of Cicer arietinum seed powder was extracted with methanol at a 1:4 (w/v) ratio and heated at 60 °C in a water bath for 1 h with constant shaking. The extract was centrifuged at 10,000 rpm for 20 min at 4 °C, filtered, and concentrated using a rotary evaporator. The concentrated extract was freeze-dried using a LABCONCO lyophilizer (Shell Freeze System, England, UK) at −40 °C, yielding a final dry extract of 15.833%. Before use, the extract was reconstituted in distilled water.

### Induction of Ehrlich Solid Carcinoma model

Ehrlich Ascites Carcinoma (EAC) cells were harvested from the peritoneal cavity of tumor-bearing mice (obtained from the National Cancer Institute (NCI), Cairo University, Egypt) using a sterile disposable syringe. EAC cells were washed with normal saline and centrifuged. Then, cell counts were determined microscopically, and cell viability was assessed using 0.4% trypan blue dye exclusion. To induce ESC tumor formation, 2 × 10⁶ viable EAC cells suspended in 0.2 ml of saline were injected intramuscularly into the right hind thigh of mice. Tumor formation was observed seven days post-inoculation.

### Experimental animals

Female Swiss albino mice were obtained from the National Research Center (NRC), aged 6–8 weeks old and weighing 25 ± 5 g, Cairo, Egypt. They were housed in polypropylene cages with wire-mesh flooring under standardized environmental conditions, including a 12-hour light-dark cycle and a maintained temperature of 22 ± 2 °C. The mice had free access to a standard diet and water, and mice were fed on a standard chow diet as described by the Association of Official Analytical Chemists, AOAC (22) [38]. After a one-week acclimation, they were randomly assigned to six groups of six mice each. This study followed the guidelines outlined in the Guide for the Care and Use of Laboratory Animals (8th edition). It was ethically approved by the Institutional Animal Care and Use Committee (IACUC) (CUFS/F/PHY/44/18) of the Faculty of Science, Cairo University, Egypt. On day 0, thirty-six mice were randomly divided into two groups: a control group (*n* = 6) that received distilled water and served as a negative control and tumor- inoculated group (*n* = 30). On day 1, the ESC-bearing mice were further divided into subgroups: one receiving distilled water (positive control, ESC), and the three groups were treated with CAE at doses of 250, 500, and 750 mg/kg b.wt. On day 7, after tumor development, CAE (750 mg/kg) was administered to an additional group as a post-treatment group. The doses of CAE were selected based on previous studies and given by gastric gavage [16, 39]. The experimental period continued for 14 days, as demonstrated in Table 1. Tumor growth was monitored every three days by measuring solid tumor volume using a Vernier digital caliper. The calculation followed the formula A × B² × 0.5, where A is the maximum diameter and B is its perpendicular dimension [40]. All mice were euthanized on day 15 of tumor inoculation by cervical dislocation under anesthesia using sodium pentobarbital (100 mg/kg), according to the Euthanasia guidelines, and as described before [36, 41–43]. The tumor mass was excised, weighed, and sectioned into three portions: one was fixed in buffered formalin and processed for immunohistochemical analysis, another was homogenized in ice-cold (0.1 M Tris-HCl) to determine the oxidative/antioxidative markers, and the third was utilized for molecular assays such as qPCR.

**Table 1:** Experimental animal grouping and treatment protocol

Days Groups	Day 0	Day 1- Day 7	Day 7- Day 14
Control	Dist. H_2_O	Dist. H_2_O	Dist. H_2_O
ESC	ESC implantation	Dist. H_2_O	Dist. H_2_O
ESC + CAE (250 mg/kg b.wt)	ESC implantation	CAE (250 mg)	CAE (250 mg)
ESC + CAE (500 mg/kg b.wt)	ESC implantation	CAE (500 mg)	CAE (500 mg)
ESC + CAE (750 mg/kg b.wt)	ESC implantation	CAE (750 mg)	CAE (750 mg)
ESC + CAE (750 mg/kg b.wt) Post-treatment	ESC implantation	Dist. H_2_O	CAE (750 mg)

*ESC* Ehrlich solid carcinoma, *CAE *Cicer arietinum extract

### Histopathological examination

After euthanasia, tumor-bearing thigh muscles were carefully dissected, rinsed with ice-cold saline, and immediately fixed in 10% neutral buffered formalin for 48 h. Tissues were then dehydrated through a graded ethanol series, cleared in xylene, and embedded in paraffin wax. Sections of 5 μm thickness were cut using a microtome. For histopathological evaluation, sections were mounted on glass slides, deparaffinized in xylene, and rehydrated through descending grades of ethanol to distilled water. Slides were stained using the standard hematoxylin and eosin (H&E) protocol [44, 45]. Hematoxylin staining for 5 min. Rinsing in running tap water. Differentiation in 1% acid alcohol, then alkaline water. Staining with eosin for 2 min. Dehydration, clearing, and mounting with DPX. Stained slides were examined using a light microscope with a magnification of 400X, and representative photomicrographs were captured for all groups.

### Oxidative/antioxidative stress markers

The level of malondialdehyde (MDA) resulting from the lipid peroxidation of ESC was measured by determining the concentration of thiobarbituric acid-reactive substances (TBARS). In addition, total antioxidant capacity (TAC), and the contents of superoxide dismutase (SOD), reduced glutathione (GSH), catalase (CAT), glutathione reductase (GR), and glutathione peroxidase (GPx) were also spectrophotometrically measured following the kit guidelines (Biodiagnostic, Egypt) as we described before [46].

### Quantitative real-time PCR (qPCR)

About 50 mg of tumor tissues were used for RNA extraction using the Qiazol lysis reagent (Qiagen, Hilden, Germany) and an RNA extraction kit (Cat #K0731, Thermo Scientific, Vilnius, Lithuania), following the manufacturer’s instructions, as described before [47]. RNA concentration and purity were assessed at 260 and 280 nm using an Infinite^®^200 PRO NanoQuant spectrophotometer (Tecan, Zürich, Switzerland). Next, 1 µg of RNA was converted into complementary DNA (cDNA) using a cDNA synthesis kit (Cat #K1622, Thermo Scientific), following the manufacturer’s instructions. The gene mRNA expression levels were quantified using the SYBR Green PCR master mix (Cat #K0251, Thermo Scientific) on a Step-One-Plus real-time PCR system (Applied Biosystems Inc., Foster City, CA, USA). The relative quantification of mRNA expression was conducted using the 2^−∆∆Ct method, as described previously [48, 49], with *Actb* serving as the housekeeping gene. The primer sequences used are depicted in Table 2 and were synthesized by Vivantis Technologies (Selangor, Malaysia).

**Table 2:** Primer sequences used for quantitative real-time PCR

Gene	Forward Primer (5’ → 3’)	Reverse Primer (5’ → 3’)
*Casp3*	GGAGTCTGACTGGAAAGCCGAA	CTTCTGGCAAGCCATCTCCTCA
*Gpx4*	CTCCATGCACGAATTCTCAG	ACGTCAGTTTTGCCTCATTG
*Gsdmd*	GGTGCTTGACTCTGGAGAACTG	GCTGCTTTGACAGCACCGTTGT
*Actb*	GCAGGAGTACGATGAGTC CG	ACGCAGCTCAGTAACAGTCC

### Immunohistochemical staining of survivin

Survivin immunohistochemical detection was performed on paraffin-embedded tissue sections of 4 μm-thickness as we previously described [50]. Slides were deparaffinized and rehydrated, and antigen retrieval was performed in citrate buffer (pH 6.0) at 95 °C for 30 min. After blocking 3% hydrogen peroxide and 1% BSA/PBS, slides were Immunostained overnight at 4 °C with an anti-survivin antibody (1:100, Santa Cruz Biotechnology), followed by a 30-minute incubation with HRP-Rabbit/Mouse (DAKO EnVision+). Nuclei were counterstained with hematoxylin, mounted with Permount^®^, and visualized. Negative controls were prepared by replacing the primary antibody with PBS.

### Statistical analysis

Statistical analysis was performed using SPSS software (version 25). The normality of data distribution was assessed by evaluating skewness and kurtosis values, confirming that all datasets were normally distributed. Accordingly, parametric tests were employed throughout the analysis. One-way and two-way ANOVA followed by Tukey’s post hoc test was used for multiple group comparisons. Data are presented as mean ± SEM; a *P*-value < 0.05 was considered statistically significant. Graphs were generated using GraphPad Prism 8.

## Results

### Antitumor effect of CAE against ESC

**Fig. 1:**
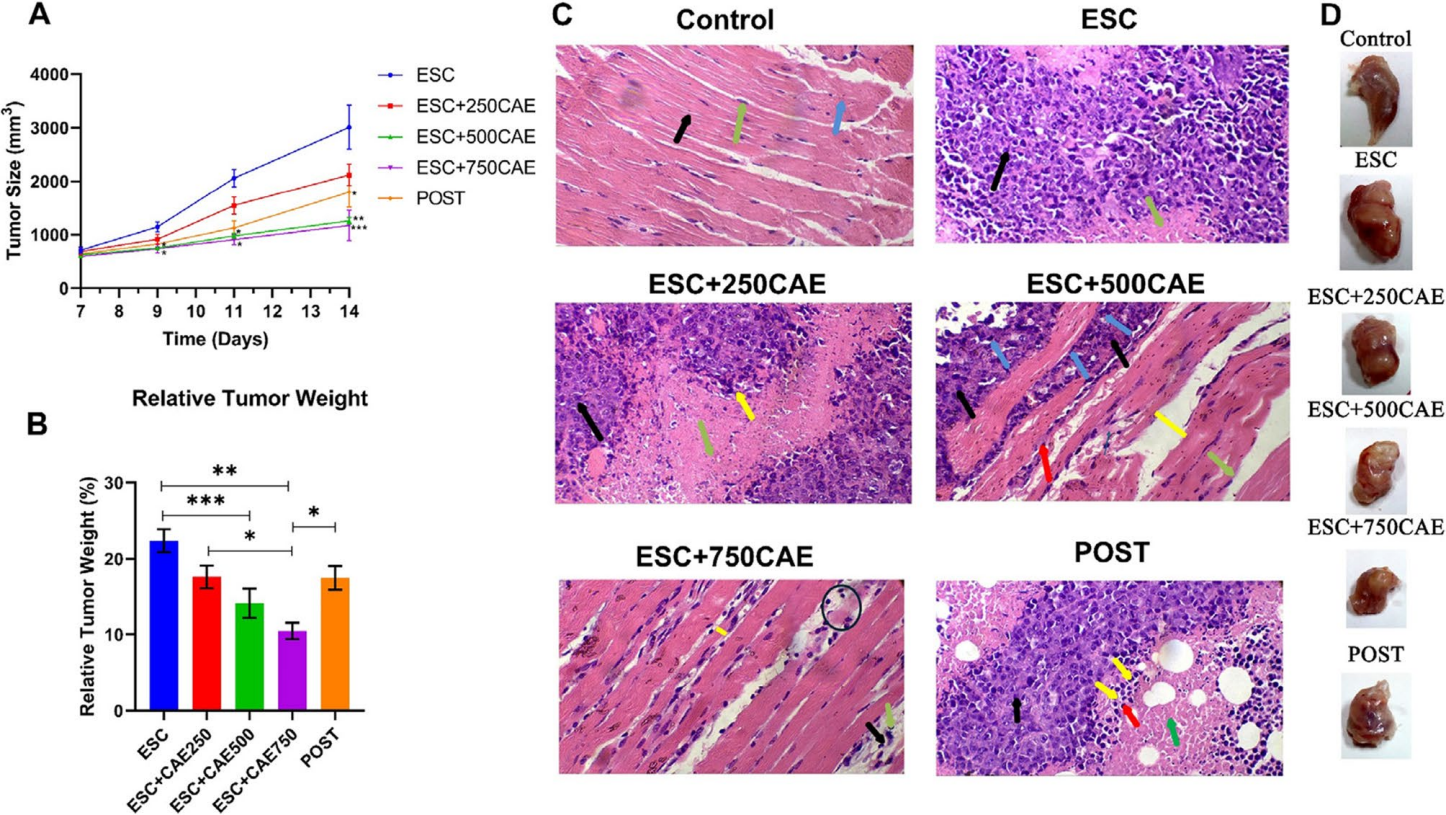
CAE suppresses tumor growth in ESC-bearing mice. (**A**) Tumor growth curve showing the mean tumor volume (mm³) over time (days 7–14) in untreated ESC-bearing mice (ESC) and in mice treated with CAE at different doses (250, 500, or 750 mg/kg) as well as post-treatment with 750 mg/kg CAE. (**B**) Bar chart showing relative tumor weight (%) across groups. A significant reduction in tumor weight was observed in all CAE-treated groups compared to the ESC group. (**C**) H&E images of the skeletal thigh muscle excised from control, untreated ESC-bearing mice and ESC-bearing mice subjected to pre-treatment with different concentrations of CAE or post-treatment with 750 mg/kg CAE (magnification ×400). Representative photos of tumor-bearing skeletal thigh muscle from each group: Control: Normal skeletal muscle architecture with intact myofibrils (black arrow), visible cross striations (blue arrow), and peripheral nuclei (green arrow). ESC: Dense infiltration of neoplastic cells with high nuclear-to-cytoplasmic ratio and pleomorphism (black arrow), and areas of necrosis (green arrow). ESC + 250CAE: Marked reduction in viable tumor tissue (black arrow), with increased necrotic areas (green arrow) and presence of apoptotic cells (yellow arrow). ESC + 500CAE: Tumorous tissue infiltration reduced (black arrow), with numerous apoptotic bodies (blue arrows), mild interstitial edema (yellow band), mononuclear cell infiltration with minimal fibrosis (red arrow), and wavy myofibrils (green arrow). ESC + 750CAE: Muscle bundles show focal atrophy (yellow band), fragmentation and degeneration (black circle), interstitial edema (black arrow), and mononuclear infiltration (green arrow). POST: Residual tumorous tissue (black arrow), abundant apoptotic bodies (yellow arrows), lymphocytic infiltrates (red arrows), and necrotic regions (green arrow). (**D**) Representative images showing the gross morphology of the whole skeletal muscle with tumor mass. Data are shown as mean ± SEM (*n* = 5), *P < 0.05, **P < 0.01, ***P < 0.001 as determined by one-way ANOVA followed by Tukey’s test

The tumor size (mm³) and relative tumor weight (%) decreased significantly at the 500 mg/kg dose by 58.2% (*P* < 0.01) and 36.8% (*P* < 0.001), and at the 750 mg/kg dose by 61% and 53.2% respectively (both *P* < 0.001), compared to the ESC group. Additionally, the post-treatment group demonstrated a significant reduction in tumor size by 40% (*P <* 0.05) versus the ESC group (Fig. 1A and B). In addition, Two-way ANOVA revealed a statistically significant effect of time (*P <* 0.001), treatment (*P <* 0.001), and the interaction between time and treatment (*P <* 0.001), indicating that tumor growth varied significantly over time and among the different treatment groups.

A histopathological examination of the skeletal thigh muscle of all groups was performed. The control group showed the normal histological structure of the muscle fibers. The ESC group revealed a massive tumor tissue that occupied most of the muscle, composed of irregular clusters of intact anaplastic cells that exhibited large, round, polygonal, and pleomorphic features with hyperchromatic nuclei. In contrast, in a dose-dependent manner, the pre-treatment groups displayed a gradual reduction of tumor tissue with partial structural integrity of muscle fibers. Similarly, the post-treatment group compared to the ESC group exhibited a significant regression of tumor tissue with the presence of widespread necrotic areas and abundant apoptotic bodies (Fig. 1C). Representative images of the gross morphology for thigh skeletal muscle without and with ESC tumors of all groups were captured, showing a noticeable reduction in tumor size of all treatment groups compared to the untreated mice group (ESC group) (Fig. 1D). These results highlight the effectiveness of CAE in hindering tumor progression in both pre- and post-treatment regimens, particularly at high doses.

### Ameliorative effect of CAE against oxidative stress in ESC tumor cells

**Fig. 2:**
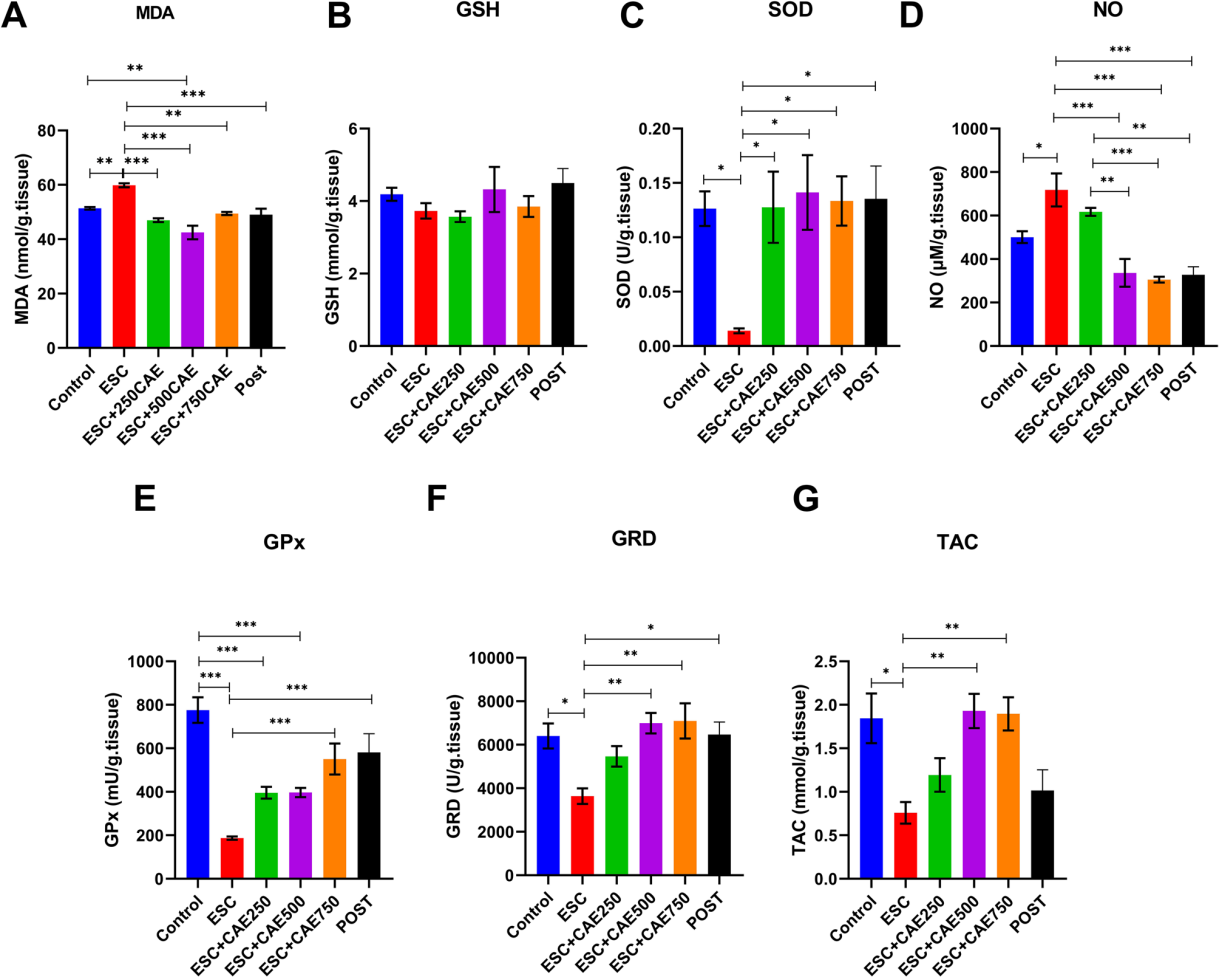
Effect of CAE on the oxidant/antioxidant status in control and ESC-bearing mice. Levels of Malondialdehyde (MDA) (**A**), Glutathione (GSH) (**B**), Superoxide dismutase (SOD) (**C**), Nitric oxide (NO) (**D**), Glutathione peroxidase (GPx) (**E**), Glutathione reductase (GRD) (**F**), and total antioxidant capacity (TAC) (**G**) were evaluated. Data are shown as mean ± SEM (*n* = 5), *P < 0.05, **P < 0.01, ***P < 0.001 as determined by one-way ANOVA followed by Tukey’s test

We assessed the impact of CAE on oxidants and antioxidant enzyme activities in mice with ESC using various biochemical tests **(**Fig. 2). In the ESC group, a significant increase in MDA levels, indicating higher lipid peroxidation (*P <* 0.01). However, treatment with CAE significantly reduced MDA levels across all doses: 250 mg/kg (*P <* 0.001), 500 mg/kg (*P <* 0.001), and 750 mg/kg (*P <* 0.01), as well as in the post-treatment group (*P <* 0.001) (Fig. 2A). Regarding the GSH, the ESC group showed a notable reduction in GSH levels; however, no significant change was observed upon CAE treatment (Fig. 2B). SOD activity was significantly reduced in the ESC group (*P <* 0.05). CAE treatment significantly increased SOD activity at all doses: 250 mg/kg (*P <* 0.05), 500 mg/kg (*P <* 0.05), and 750 mg/kg (*P <* 0.05) (Fig. 2C). NO levels were also elevated in the ESC group (*P <* 0.05), but CAE treatment significantly decreased these levels at all doses (*P <* 0.001) except for 250 mg/kg which showed no significant decrease, furthermore, a significant reduction in NO level was observed in 500 mg/kg (*P* < 0.01), and 750 mg/kg (*P* < 0.001), as well as in the post-treatment group (*P* < 0.01) compared to the 250 mg/kg group (Fig. 2D). GPx activity was substantially lower in the ESC group (*P <* 0.001), with significant increases observed in CAE-treated groups: 750 mg/kg (*P <* 0.001), and Post (*P <* 0.001) (Fig. 2E), although the increase didn’t reach the levels of control group as demonstrated by a significant decrease in GPx activity in CAE treatment groups 250 mg/kg (*P <* 0.001), and 500 mg/kg (*P <* 0.001) compared to the control group. Interestingly, GPX increased significantly in the 750 mg/kg CAE and post-treated groups compared to the ESC group, without a significant difference from the control group (*P* > 0.05). In addition, GRD activity was reduced in the ESC group (*P <* 0.05), but significantly increased with CAE treatment at doses of 500 mg/kg (*P <* 0.01) and 750 mg/kg (*P <* 0.01), and POST group (*P <* 0.05) (Fig. 2F). TAC showed a decrease in the ESC group (*P <* 0.05), but CAE treatment significantly improved TAC at doses of 500 mg/kg (*P <* 0.01) and 750 mg/kg (*P <* 0.01) (Fig. 2G). These findings suggest that CAE treatment effectively alleviates oxidative stress and enhances antioxidant defenses in ESC-bearing mice, contributing to reduced tumor growth that could be induced *via *apoptosis. Thus, we quantified the expression levels of apoptosis- and pyroptosis-related genes. 

### CAE modulates the expression of antioxidant (*Gpx4*), apoptosis (*Casp3*)-, and pyroptosis-related gene (*Gsdmd*) in ESC tumors

**Fig. 3:**
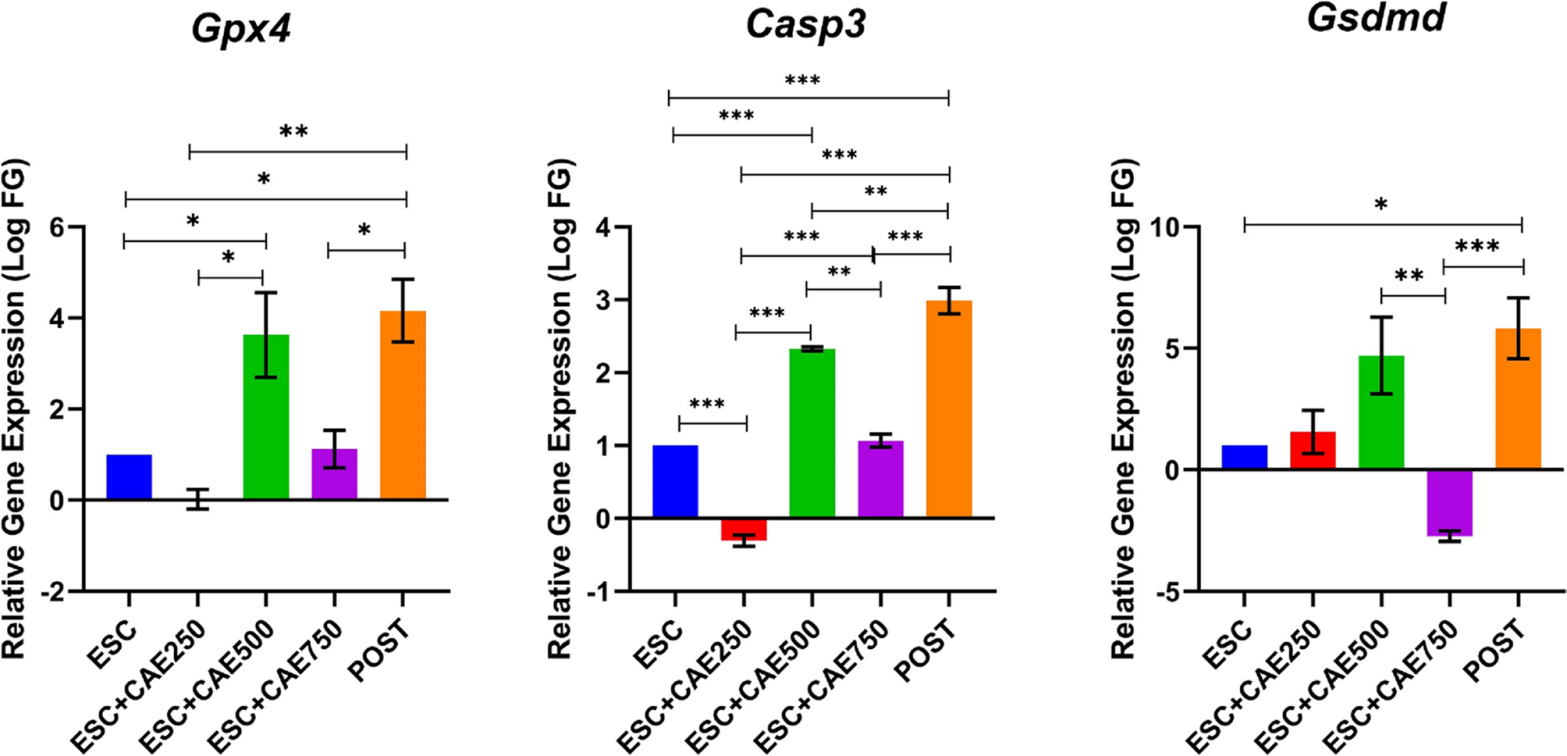
The mRNA expression level of antioxidant (*Gpx4*), pro-apoptotic gene (*Casp3*), and pyroptosis-related gene (*Gsdmd*). The mRNA expression levels of the antioxidant gene (*Gpx4*) (left panel). The mRNA levels of pro-apoptotic genes *Casp3* (middle panel) in ESC-bearing mice. The mRNA expression level of the pyroptosis-related gene (*Gsdmd*) (right panel). The groups include ESC (Ehrlich Solid Carcinoma), ESC + CAE (*Cicer arietinum* extract) pre-treatment at different doses (250 mg/kg, 500 mg/kg, and 750 mg/kg), and POST (post-treatment group). Data are shown as mean ± SEM (*n* = 5), *P < 0.05, **P < 0.01, ***P < 0.001 as determined by one-way ANOVA followed by Tukey’s test

Next, we assessed the mRNA expression levels of the antioxidant-related gene (*Gpx4*), apoptosis-related gene (*Casp3*), and pyroptosis-related gene (*Gsdmd*). *Gpx4* expression was significantly upregulated in the CAE 500 mg/kg group and the post-treatment group (*P* < 0.05) compared to the ESC group (*P <* 0.05). The post-treatment group also showed significantly higher *Gpx4* levels than the CAE 250 mg/kg group (*P <* 0.01) and the CAE 750 mg/kg group (*P <* 0.05). In addition, the CAE 500 mg/kg group showed a significant increase (*P* < 0.05) compared to the CAE 250 mg/kg group. *Casp3* expression was markedly elevated in the CAE 500 mg/kg and post-treatment groups compared to the ESC (*P <* 0.001), the CAE 250 mg/kg group (*P <* 0.001), and the CAE 750 mg/kg group (*P <* 0.01). Furthermore, the CAE 750 mg/kg group showed a significantly higher *Casp3* expression than the CAE 250 mg/kg group (*P <* 0.001). Notably, the post-treatment group exhibited the highest expression, compared to the ESC (*P <* 0.001), the CAE 500 mg/kg group (*P <* 0.01), and the CAE 750 mg/kg group (*P <* 0.001). *Gsdmd* expression was significantly upregulated in the post-treatment group compared to ESC (*P <* 0.05), and CAE 750 mg/kg (*P <* 0.001). Conversely, the CAE 750 mg/kg group demonstrated a significant downregulation compared to CAE 500 mg/kg (*P <* 0.001) (Fig. 3).

### CAE downregulates survivin expression

**Fig. 4:**
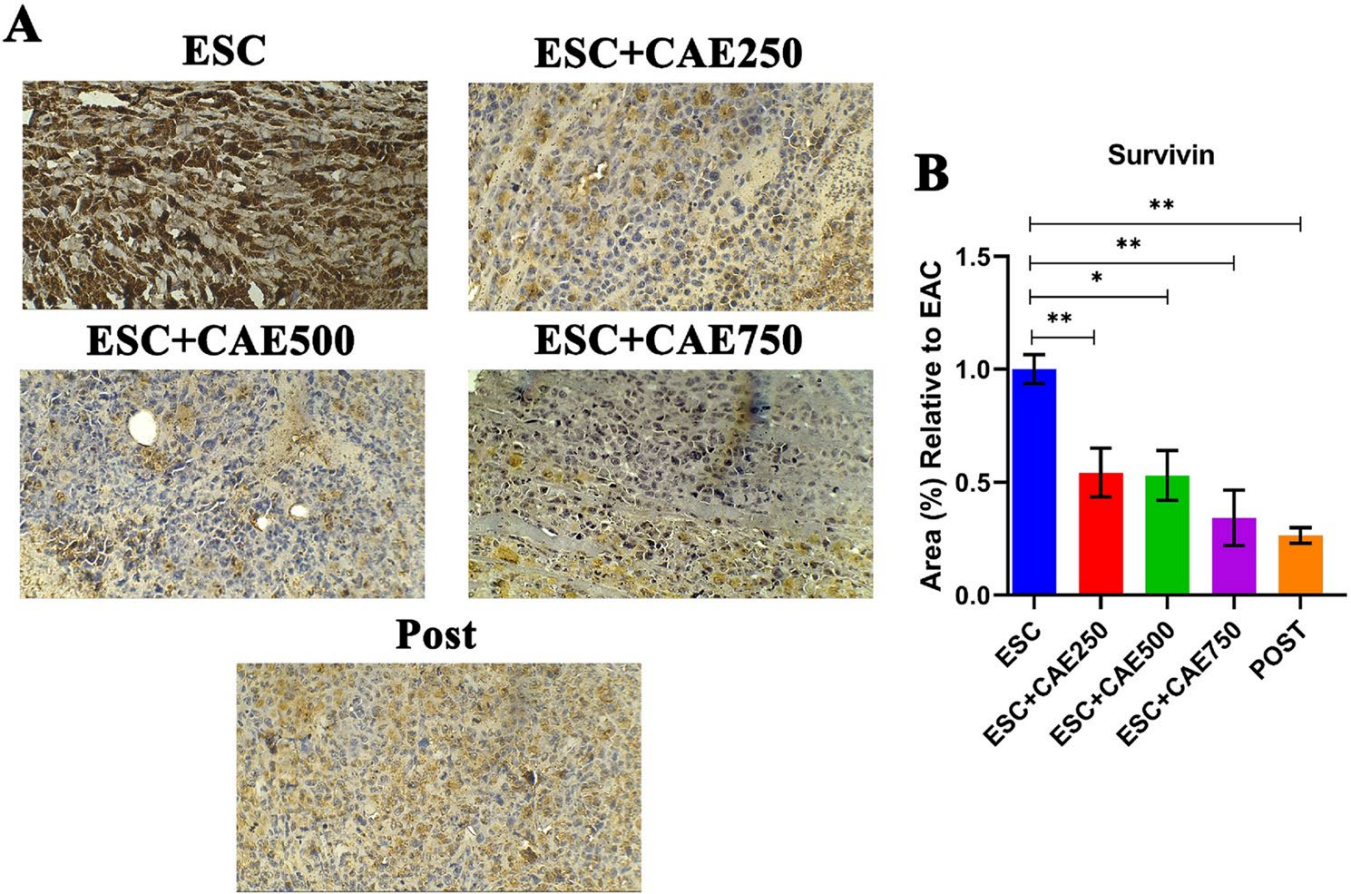
Immunohistochemical Analysis of Survivin Expression in Different Treatment Groups. (**A**) Representative immunohistochemical staining for Survivin in tissue sections from different treatment groups. (**B)** Quantitative analysis of Survivin-positive area (%) relative to the ESC group. The ESC group exhibits the highest Survivin expression, as indicated by the extensive brown staining, which significantly decreases in the CAE pre- and post-treated groups. Data are shown as mean ± SEM (*n* = 5), *P < 0.05, **P < 0.01, ***P < 0.001 as determined by one-way ANOVA followed by Tukey’s test

Since survivin is a member of the inhibitor of apoptosis protein family and has a crucial role in cancer cell survival [51], we examined its expression pattern as a clue for the antitumor effect of CAE in the studied groups using IHC. IHC revealed that untreated ESC-bearing mice exhibited prominently elevated immunostaining of survivin, while CAE treatment resulted in significant repression of survivin expression in a dose-dependent manner, specifically, at a dose of 750 mg/kg, administered either pre- (*P <* 0.05) or post-treatment (*P <* 0.01) (Fig. 4).

### CAE treatment shows no signs of toxicity and effectively hinders ESC metastasis to vital organs

**Fig. 5:**
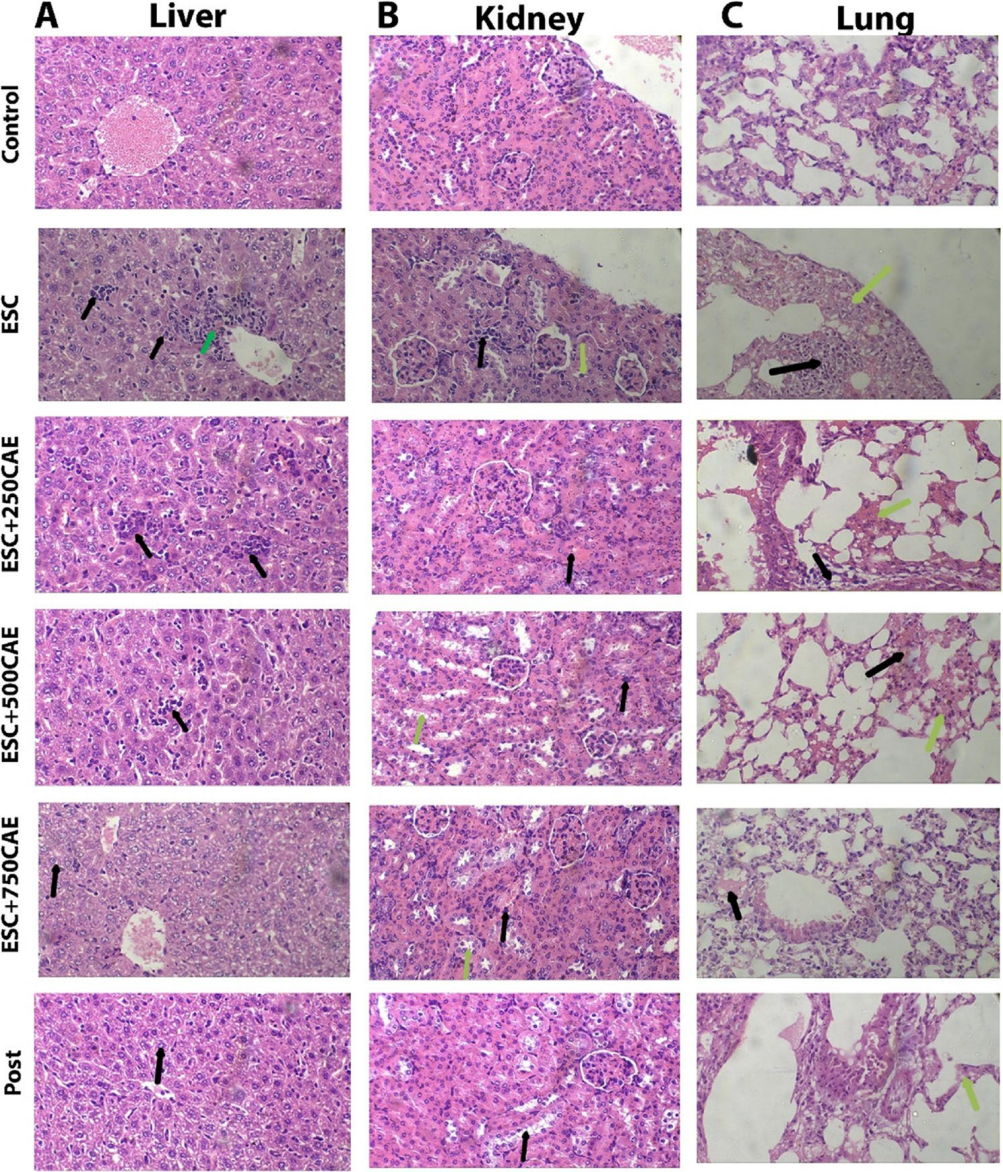
Histological sections of liver, kidney, and lung tissues from different experimental groups (H&E stain, 400x). (**A**) Representative images of the liver section: control samples showed uniform hepatocyte architecture. In the ESC group, aggregates of neoplastic cells (black arrows) and focal hepatocyte necrosis (green arrows) were observed. The 250 mg/kg CAE group showed persistent neoplastic aggregates (black arrows), while the 500 mg/kg group displayed few clusters of apoptotic neoplastic cells. No neoplastic cells were detected at 750 mg/kg and in the post-treatment group; however, mild hydropic changes of hepatocytes were noted (black arrows) in both. (**B**) Representative images of the kidney: ESC kidneys, showed neoplastic cell collections (black arrows) and mild hydropic degeneration (green arrows). At 250 mg/kg CAE, mild hydropic degeneration was present (black arrows). Both 500 mg/kg and 750 mg/kg groups showed residual apoptotic neoplastic cells (black arrows) along with mild hydropic degeneration (green arrows). Post-treatment kidneys had no neoplastic cells, with mild tubular hydropic changes still evident. (**C**) Representative images of the lung: ESC lungs exhibited malignant cell infiltration (black arrows), congestion, and inflammatory infiltrate (green arrows). At 250 mg/kg CAE, apoptotic neoplastic cell aggregates (black arrows) were present along with congestion and inflammation (green arrows). Lungs from the 500 mg/kg group showed mild interstitial pneumonia with septal thickening, inflammatory infiltrate (green arrows), and focal congestion (black arrows), with no detectable neoplastic cells. Similar findings were seen at 750 mg/kg, along with minimal pulmonary edema (black arrows). Post-treatment lungs showed an inflammatory infiltrate (green arrows) without any detectable neoplastic cells

Histopathological examination of the liver, kidney, and lung tissues from CAE-treated mice demonstrated no significant toxicological alterations across all tested doses. In hepatic sections, normal architecture was preserved in the control group, while the untreated ESC group exhibited aggregates of neoplastic cells accompanied by focal hepatocyte necrosis. Administration of CAE at 250 mg/kg reduced the extent of neoplastic infiltration, though small clusters of tumor cells were still detectable. At doses of 500 mg/kg, only a few apoptotic neoplastic clusters remained, whereas both the 750 mg/kg and post-treatment groups showed a complete absence of neoplastic cells, indicating the anti-metastatic effect of CAE on hepatic dissemination. No signs of necrosis, cholestasis, fibrosis, or remarkable inflammatory infiltration in hepatocytes were observed (Fig. 5A).

In renal tissues, the control group showed preserved histology. In contrast, the untreated group exhibited prominent neoplastic infiltration and tubular degeneration. Treatment with CAE at 250 mg/kg led to the reduction of tumor burden, with no viable neoplastic cells observed in the post-treatment group, and only residual apoptotic tumor cells remaining in the 500 and 750 mg/kg groups. These findings reflect a dose-dependent reduction of metastatic involvement in the kidney. The accompanying tubular hydropic changes were mild and reversible, with no evidence of tubular necrosis, tubulitis, interstitial inflammation, or glomerular damage (Fig. 5B).

Lung tissue analysis of the untreated ESC group showed widespread malignant infiltration, congestion, and inflammatory response. CAE treatment led to a progressive clearance of neoplastic cells from lung tissues. At 250 mg/kg, apoptotic tumor clusters were still apparent, but by 500 mg/kg and higher doses, no viable neoplastic cells were detected. Post-treatment groups also demonstrated a complete absence of neoplastic infiltration, indicating that CAE not only mitigated primary tumor burden but also effectively suppressed pulmonary metastasis. Histological changes such as interstitial inflammation and septal thickening were mild (Fig. 5C).

Collectively, these histopathological findings confirm the dual benefit of CAE administration: a favorable safety profile with no significant toxicity to vital organs and a clear suppressive effect on the metastatic spread of ESC cells, particularly evident at higher doses and in post-treatment groups.

## Discussion

Developing safe and effective cancer therapies remains one of the greatest challenges in the field of cancer research. Accumulating evidence highlights the impact of nutrition on the onset and progression of various cancers [52, 53]. This has sparked a growing interest in understanding the impact of natural phytochemical extracts as a safe and promising approach to cancer prevention and treatment [54, 55].

CAE is recognized as a rich source of diverse phytochemical compounds, including isoflavones, which function as natural chemoprotective agents against cancer [12]. Previously, we investigated the impact of CAE in vitro, observing its inhibitory effect on breast cancer cells that could be mediated *via* attenuation of the p44/42 MAPK signaling pathway [17]. Based on our previous work, the current study expands the scope of CAE’s antitumor potential by evaluating its effects in a murine model of ESC. Currently, we showed that pre- and post-treatments significantly reduced tumor growth, downregulated ROS levels, enhanced antioxidant defense mechanisms, and suppressed survivin and elevated *Casp3* expressions. Additionally, the post-treatment strategy significantly induced *Gsdmd* gene expression.

Most of the previous studies analyzed the effect of isolated specific groups of CAE on cancer development; however, research on the protective and therapeutic effects of whole CAE, containing all its bioactive compounds, on cancer initiation and progression in vivo is limited. For instance, Giron-Calle et al. observed that a peptide fraction derived from chickpea concentrate demonstrated an 80% reduction in the proliferation of CaCo-2 colon cancer cells following a 4–6-day treatment [56]. Additionally, the effect of protease inhibitors extracted from chickpeas demonstrated anticancer properties in breast and prostate cancer cell lines, leading to a reduction in cell proliferation by 12–14% and 32–37%, respectively [25]. Besides, hydrophilic extracts derived from chickpea flour demonstrated anti-proliferative effects against SW480 colon cancer cells [57]. Furthermore, it has been observed that suppression of β-catenin nuclear translocation in colon cancer cells in an AOM/DSS mouse model occurs when fed a diet containing 10% chickpea [58], while a 20% chickpea-containing diet significantly induced downregulation of proliferative markers and attenuation of inflammation, leading to tumor reduction [31]. Collectively, both 10% and 20% chickpea diets exhibited chemo-preventive effects. Moreover, a virtual screening study for 1720 phytochemicals from 29 traditional Egyptian medicinal plants was conducted to identify the potential of natural human aromatase enzyme inhibitors against hormone-dependent breast cancer, revealing an inhibitory effect of *Cicer arietinum* towards this molecular subtype of breast cancer [59].

Although HPLC profiling was not conducted in the current study, the extract was prepared and characterized using the same method as described in our previous publication (Fahmy et al., 2015), where isoflavones, including Formononetin, genistein, biochanin A, and daidzein, were identified. Reactive oxygen species (ROS) are considered oncogenic signaling molecules since they contribute to cancer initiation, progression, invasion, and metastasis through various signaling pathways [60]. Oxidative stress arises from an imbalance between pro-oxidant and antioxidant mechanisms, leading to overproduction of ROS [61–63]. Oxidative stress has an important role in maintaining cancer cell survival and proliferation [64–66]. Herein, CAE pre- and post-treatments significantly decreased MDA levels, while enhancing the activities of ROS scavenging enzymes, including SOD, CAT, GPx, GRD, and elevated TAC in tumor tissues, and enhanced the expression of the antioxidant enzyme *Gpx4* at the mRNA levels. Consistently, our previous findings showed that CAE has hydrogen peroxide (H_2_O_2_) and superoxide radical (O_2_^**·**^) scavenging activities, leading to reduced oxidative damage stimulated by γ-irradiation in various organs [46]. These findings suggest that CAE enhances tumor growth retardation *via* the enhancement of different antioxidant defense molecules that, in turn, reduce ROS levels.

Similarly, antioxidant compounds, such as butein, genistein, tea polyphenols, and pyrrolidine dithiocarbamate, can suppress breast and prostate cancer cell growth *via* ROS level reduction [67], GPx activation [68], and survivin inhibition [69, 70]. Moreover, it has been identified that six isoflavones in sprouted black chickpea extract exhibited potential antioxidant activity associated with antiproliferative effects toward breast cancer cells [28]. Similarly, *Cicer arietinum L*. sprouts extract demonstrated the most substantial reduction in intracellular oxidative stress in human osteosarcoma Saos-2 and human breast cancer MCF-7 cell lines. Additionally, elevated concentrations of CAE induce apoptosis in estrogen-dependent MCF-7 cells [71]. Taken together, these findings indicate that dietary supplementation with combined bioactive compounds that naturally exist in seeds or leaves of plants may play a crucial role in the treatment of cancer and other pathological conditions.

Previous studies have showen that survivin expression positively regulates tumor growth in various carcinomas both in vivo and in vitro, including colorectal cancer [72], breast cancer [73], prostate cancer [74], and melanoma [75]. It is worth mentioning that survivin plays a central role in the production of cellular energy and ROS via maintaining a mitochondrial oxidative phosphorylation system and inhibiting apoptosis [76, 77, 78, 79]. In turn, ROS production induces survivin expression *via* NF-κB activation in cancer cells [80]. Using natural compounds such as Gigantol [81], Sanguinarine [74], and Diindolylmethane [82], it has been shown effectively reduced breast and prostate tumor formation through survivin downregulation. Thus, in light of the evidence mentioned above, we speculate that the attenuated expression of survivin in the tumor tissue of CAE-treated groups contributed to tumor growth inhibition and enhanced sensitivity to apoptosis

Caspase-3, a key effector in apoptosis, typically cleaves and activates downstream apoptotic proteins, leading to cell death through controlled, non-inflammatory processes [83, 84]. Chickpea-derived lectin, peptides, and isoflavones exhibit significant anticancer effects through the induction of cell cycle arrest and apoptosis *via* activating caspases, including caspase-3, caspase 7, and caspase 9, and the upregulation of the tumor suppressor genes P53 and P21 in a dose-dependent manner in breast and endometrial cancer cells [24, 29, 30, 85]. Pyroptosis, a distinct type of programmed cell death associated with inflammatory responses, has emerged as a novel cancer therapeutic strategy for its ability to overcome chemoresistance [86]. Pyroptosis is primarily triggered by inflammasome activation, which activates caspase enzymes, such as caspase-1, −3, −4, −5, −8, and − 11). These caspases mediate the cleavage of gasdermin proteins, resulting in pore formation within the plasma membrane and compromising its structural integrity [87–90]. A key member of the gasdermin protein family is gasdermin D (GSDMD), which plays a central role in the classical pyroptosis pathway, upon its cleavage by active caspase-1 [91]. In the current study, we observed an upregulation of the *Gsdmd *gene expression under post-treatment conditions ,suggesting the potential involvement of an alternative type of cell death, distinct from apoptosis, in ESCs following treatment. The switch between apoptosis and pyroptosis is regulated by different signaling pathways and proteins, including gasdermin proteins (76). Aligning with our results, recent studies have reported that natural compounds, such as Reniformin A, Cinobufotalin, Omega-3 docosahexaenoic acid, metformin, and anthocyanin, induce tumor regression *via* the induction of GSDMD-mediated pyroptosis in various cancers [86, 92–95]. Thus, according to the evidence discussed above, we hypothesize that the bioactive component of CAE triggers both apoptotic and pyroptotic cell death following post-treatment.

In the present study, we observed a significant reduction in ESC growth following both pre- and post-treatment with moderate and higher doses of CAE. These chemopreventive and antitumor effects are presumed to be facilitated by the attenuation of oxidative stress and survivin expression, as well as the induction of apoptosis. Additionally, pyroptosis appears to be involved in the antitumor treatment response. These actions are heavily reliant on CAE's their bioactive components in line of the previously described studies. We hypothesize that CAE is a promising phytochemical with both protective and therapeutic potential for cancer prevention and treatment.

Several challenges should be addressed before clinical translation of our findings. This includes the need for large-scale standardization of extract preparation, comprehensive toxicological evaluations, pharmacokinetic profiling, and isolation of specific active components. In addition, we were unable to conduct a survival analysis, as this tumor model is aggressive and fast-growing. Therefore, the study duration must be limited to three weeks from the day of tumor induction [96–98].

### Conclusion and future perspectives

**Fig. 6:**
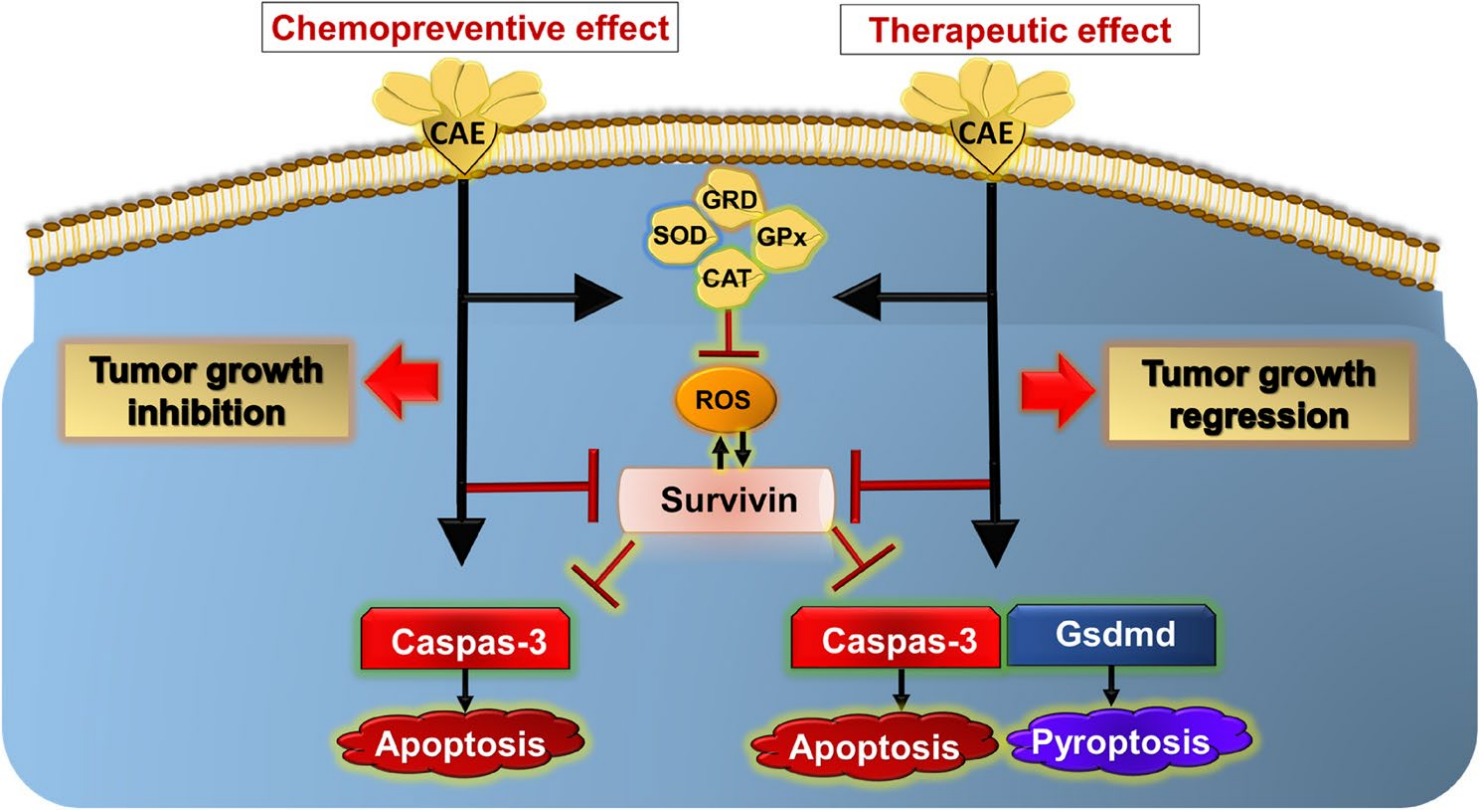
A Schematic Representation of the Molecular Mechanisms of CAE as a chemopreventive and therapeutic agent in inhibiting Tumor Growth. Survivin: CAE treatment led to a decrease in survivin levels, a protein that inhibits apoptosis, enhances ROS production, and promotes cell survival, while its reduction results in increased apoptotic sensitivity. Antioxidant Enzyme Activation: CAE increases the activity of antioxidant enzymes such as superoxide dismutase (SOD), glutathione peroxidase (GPx), catalase (CAT), and glutathione reductase (GRD). These enzymes mitigate the effects of reactive oxygen species (ROS), which can damage cellular components and contribute to oncogenic signaling. Caspase-3: The combined effects of survivin downregulation and antioxidant enzyme activation might lead to upregulation of the apoptotic marker Caspase-3. Gsdmd: CAE therapeutic effect induces a pyroptotic marker Gsdmd expression that could enhance the pyroptotic pathway as an alternative cell death pathway to apoptosis. Tumor Growth: The overall effect of these molecular changes is the suppression of tumor growth in both preventive and treatment strategies, as evidenced by the reduced size of tumors in treated mice

Overall, these findings suggest that CAE pre-treatment acts as an effective chemopreventive agent by hindering tumor growth, restoring antioxidant activity, and downregulating survivin expression to normal levels, whereas post-treatment acts as an ideal cancer therapy by inducing tumor shrinkage, upregulating *Casp3* and *Gsdmd* expression, and reducing survivin expression, and at the same time lowering the oxidative stress (Fig. 6). Thus, we postulate that CAE possesses promising chemopreventive and therapeutic potential against solid cancers.

As a future perspective, we propose formulating CAE into phytocomposite nanoparticles for direct intratumoral administration. This strategy would enhance its bioavailability and stability, while also allowing for selective targeting of cancer tissue. Furthermore, integrating artificial intelligence (AI) with molecular docking and dynamics simulations could help identify the specific target proteins that interact with the most abundant and active components in the CAE extract. These studies would provide deeper insights into the mechanism of action of the CAE and could aid in designing an enriched nano-formulation containing the most effective compounds.

## Data Availability

The datasets used and/or analyzed during the current study are available from the corresponding author upon reasonable request.

## Abbreviations

CAE: Cicer arietinum extract
CAM: Complementary and alternative medicine
Casp3: Caspase-3
CAT: Catalase
cDNA: Complementary DNA
EAC: Ehrlich Ascites Carcinoma
EGF: Epidermal growth factor
ESC: Ehrlich solid carcinoma
GPx: Glutathione peroxidase
GR: Glutathione reductase
GSH: Glutathione
H2O2: Hydrogen peroxide
IHC: Immunohistochemistry
NRC: National Research Center
ROS: Reactive oxygen species
SOD: Superoxide dismutase
TAC: Total antioxidant capacity
TBARS: Thiobarbituric acid-reactive substances
TNBC: Triple-negative breast cancer
